# Variable Thickness in Plates—A Solution for SHM Based on the Topological Derivative

**DOI:** 10.3390/s20092529

**Published:** 2020-04-29

**Authors:** Anxo Martínez, Alfredo Güemes, Jose M. Perales, Jose M. Vega

**Affiliations:** 1 Department of Applied Mathematics, Universidad Politécnica de Madrid, UPM, E-28040 Madrid, Spain; anjo.mdominguez@alumnos.upm.es (A.M.); josemanuel.vega@upm.es (J.M.V.); 2 Center of Composites Materials and Smart Structures, Universidad Politécnica de Madrid, UPM, E-28040 Madrid, Spain; alfredo.guemes@upm.es; 3 Department of Aerospace Vehicles, Universidad Politécnica de Madrid, UPM, E-28040 Madrid, Spain

**Keywords:** non-destructive testing, defect detection, structural health monitoring, guided waves, variable thickness, complex cross-section, inverse problems, multi-frequency, topological derivative

## Abstract

The topological derivative tool is applied here in structural health monitoring (SHM) problems to locate small defects in a material plate with complex geometry that is subject to permanent multifrequency guided waves excitation. Compared to more standard SHM methods, based in measuring the time-lag between emitted and received propagative pulses plus some postprocessing, the topological derivative somehow compares the measured and computed (solving the full elasto-dynamic equations) response of the damaged plate, instead of relying on only the time of flight of the wave. Thus, the method profits the knowledge behind the physics of the problem and can cope with scenarios in which classical methods give poor results. The authors of this paper have already used the topological derivative in rectangular plates with constant thickness, but with defects consisting simply in both through slits and inclusions of a different material, and actuators/sensors located near the boundary, which makes very difficult to use standard SHM methods. This is an extension of the method, also considering the much more difficult to analyze case of plates with variable thickness and complex (non-rectangular) planform.

## 1. Introduction

Over the last decades, *inverse problems* [1] have received the attention of engineers and applied mathematicians due to both their intrinsic scientific interest and their multiple applications. Inverse problems consist of determining the physical properties of a medium after its response to excitation. These problems are closely related to parameter estimation [2] and are usually involved when fault diagnosis is required (note that faults produce changes in the physical properties of the system). Inverse problems are of interest in many fields, including, for example, natural resources exploration [3], oceanography [4], medical diagnosis [5], anti-personnel landmines location [6], and non-destructive testing of structures [7]. Inverse problems can be seen as the opposite to *direct problems*, which, in turn, consist of calculating the system response when its physical properties are known. The main difficulty is that inverse problems are usually ill-posed problems [8], because the physical properties of the system do not depend continuously on its response. That is, small changes in the system response may easily produce large variations in the estimated properties of the medium. The direct problem, instead, is usually well-posed (when it is properly formulated). Besides, efficient diagnosis usually requires assessing the properties of the system using a fairly small amount of data. For instance, in ultrasonic testing of structures, the whole three-dimensional distribution (location and size) of the faults are to be guessed from measurements of the system response in just a few amount of sensors.

Inverse problems are behind all Structural Health Monitoring (SHM) techniques as they intend to detect faults, for example, defects and inclusions (which affect material parameters such as the density, the Poisson ratio or the Young modulus). In particular, SHM done by using forced guided waves [9,10] has attracted considerable attention in the field in recent years, specially to monitor structural plates. The idea behind is that faulty regions scatter the guided waves producing a pattern of waves different from that of a healthy plate. In standard SHM methods, the defects localization is determined measuring the time-lags between the emitted and received pulses. These time-lags are later converted into distances using the constant wave propagation velocity, which is known. In time-of-flight methods, it is very difficult (or simply it is not possible) to distinguish secondary echoes (those coming from behind the first fault echo, coming from the boundaries or coming from a change in acoustic impedance) from primary echoes so the field of view is handicapped. The present method, instead, can cope with all these situations. Instead of just using the ‘time of flight’, and ignore any diffraction effect, there are available more elaborate conventional methods that also take diffraction into account using what we can consider as variants of tomographic inspection [11,12,13,14,15] and sensing networks [16]. These conventional methods can be applied to several physical phenomena (e.g., elastic or electromagnetic waves) because they only rely on the propagation velocity and, thus, they are not influenced by the wave motion physics behind. One of their drawbacks is that they require appropriate location of an appropriate number of sensors [17,18], a good knowledge of the propagation velocity along the plate, and a good means to distinguish between the primary echoes scattered by the faults and secondary echoes (that may happen from the reflection at the plate boundary). Thus, they exhibit drawbacks associated with:

- The mixing between the primary emitted waves and their reflection at the side walls, that may lead to very complex wave patterns [19,20]. This can be problematic when either:
  – Actuators or sensors are near the boundaries, as this strongly mixes the reflected waves and primary waves which widens the width of the received signals.
  – The planform of the plate is curved and complex [21], which produces very complex reflected waves that may even lead to caustics [22].
- The variation of the propagation velocity in the plate when its thickness is not constant [23]. In theory, since the thickness is known, the wave propagation is known too, but accounting for the effect of the variable wave propagation in the above mentioned relation between time-lags and distances is very problematic.

The object of the present work is to use a more sophisticated mathematical method for solving these inverse problems that will be able to cope with the difficulties mentioned above. The idea is to take into account the whole physics of the system (which in the present context govern the phenomena of reflection, diffraction, and scattering of the elastic waves). Several mathematical methods to solve inverse problems can be found in Reference [24]. Some of these methods rely in formulating the inverse problem as an optimization problem, where one tries to minimize the difference between the computed (for varying values of the material properties) and the experimentally measured system responses. The guessed values of the material properties will then assumed to be those that minimize the cost function, which may be computed using an iterative method [25]. However, appealing as it is, this method either requires an appropriate starting initial condition (which, usually, necessitates an a priori knowledge of the number and the approximate position of the defects) or involves a large number of iterations. A very good alternative is the so called *topological derivative* [26,27,28], where applications can already be found in the context of SHM using either thermographies [29] or guided waves [30], and is nowadays a well established technique to solve inverse problems. In fact, topological derivative based strategies have been already successfully used to solve inverse problems in a variety of fields, including acoustic [31,32,33], elastic [34,35,36,37,38,39], electromagnetic [40,41,42,43], and thermal [44,45] waves, as well as electrical impedance tomography [46,47,48].

As will be seen below, the topological derivative concept is closely related to the value of the *sensitivity* of the above mentioned cost function to local perturbations (deviations of the measurements from the healthy results) in the material properties, which are produced by the presence faults. Thus, one can expect that localized defects are located in those regions where the sensitivity is largest, namely near the topological derivative peaks.

As already mentioned, the topological derivative has been used by us for SHM using guided waves in Reference [30], where several scenarios were considered that could hardly be treated by standard SHM methods. Namely, (a) excitation/sensing devices located close to the boundary of the plate and (b) very small faults appearing beyond elongated through slits and/or inclusions of a different material (which somewhat mimic stringers in aircraft wings). Here, we will extend the results in three different directions, by considering the scenarios (a) and (b) mixed with two new ones, namely: plates with (c) variable thickness and (d) complex planform. Thus, (e) instead of the two-dimensional approximation of the elastodynamic equations used in Reference [30], we rely on a fully three-dimensional formulation; this is because we shall deal with plates with sharp thickness variations, which cannot be modeled at all using a two-dimensional approximation.

Although the three dimensional formulation allows to deal with general Lamb waves, here we shall restrict ourselves to symmetric waves, which may be produced by symmetric in-plane excitation. This is because symmetric and anti-symmetric waves exhibit disparate phase velocities [49] at the frequencies that are usually used in guided waves excitation in SHM. This produces disparate wavenumbers that lead to multi-scale wave patterns. In addition, numerical modeling of antisymmetric Lamb waves require a very fine mesh, which increases the computational cost of the simulations. On the other hand, instead of considering propagative waves, as in standard SHM methods, here, as in Reference [30], we consider permanent multi-frequency waves. These may be produced by permanent multi-frequency excitation and identified after appropriate de-convolution.

With a proof-of-the-concept aim, we are going to use numerically computed synthetic data instead of actual experimental data. Experimental noise will be mimicked by using fairly different numerical grids for the computation of the synthetic data and for the calculation of the topological derivative. This use of different meshes also aims to avoid the so-called inverse crime phenomena in the context of solving inverse problems. Actual experimental noise could be treated using HODMD (higher order dynamic mode decomposition), a recent method [50] that also de-convolutes multi-frequency data. HODMD has already been shown to be very effective in cleaning and de-convoluting oscillatory experimental data obtained from particle image velocimetry (PIV) measurements [51], laser imaging detection and ranging (LIDAR) measurements [52] and aircraft flight tests [53]

Actuators and sensors will synthetically piezoelectric [54,55] or mimic either non-contact laser Doppler [56,57,58,59,60]. This is despite the fact that non-contact generation of Lamb waves can be difficult from the practical point of view using optical laser methods [61] or magnetostrictive techniques [62], because such non-contact generation requires high-power devices.

Let us point out that the paper will contain a non-trivial mathematical analysis, which is needed to make it self-contained, instead of just relying on various references in the literature. This has the additional advantage of allowing the reader to both fully understand the concepts and reproduce and check the analysis in the paper.

Against this background, the remainder of the paper is organized as follows. The theoretical background, including both the elastodynamic equations that will be used and the topological derivative definition and computation details will be addressed in Section 2. The main results of the paper will be considered in Section 3, where the cases of plates with variable thickness and complex planform will be dealt with. Let us mention here that, even though a large number of different cases have been considered and analyzed, for the sake of brevity only the most representative ones will be presented here. The paper ends with some concluding remarks, in Section 4.

## 2. Elastodynamic Problem and Topological Derivative

Let us first formulate the elastodynamic problem that leads to Lamb waves, in Section 2.1. Then, we consider in Section 2.2 several forcing and sensing types mimicking laser Doppler and piezoelectric devices. The above mentioned cost function is defined in Section 2.3 and its sensitivity to perturbations of the material properties, in Section 2.4. The topological derivative is defined in Section 2.5, where its computation is also summarized.

### 2.1. Formulation of the Elastodynamic Problem

Let us consider a thin plate with a not necessarily rectangular planform Γ. For simplicity, we consider a flat plate, though the analysis below also applies to more general plate shapes. Using a Cartesian coordinate system with the *x* and *y* axes in the mid-plane of the plate, the three-dimensional domain occupied by the plate is defined as

(1) $$\Omega :\;\left(x,y\right)\in \Gamma \;\mathrm{and}\;-\frac{h(x,y)}{2}\leq z\leq \frac{h(x,y)}{2},$$

where the (variable) thickness of the plate h(x,y) is small compared to the dimensions of Γ. The boundary of the domain Ω, denoted hereinafter as ∂Ω, is given by

(2) $$\partial \Omega :\;\left(x,y\right)\in \partial \Gamma \;\mathrm{or}\;z=\pm \frac{h(x,y)}{2},$$

where ∂Γ denotes the boundary of the plate planform Γ. In the applications considered in this paper, Γ will either be rectangular or complex shaped.

To the linear approximation, assuming that the *displacement vector* $\vec{u}$ is conveniently small, the *stress tensor* is defined as $\bar{\bar{\sigma }}=\lambda \left(\nabla \cdot \vec{u}\right){\bar{\bar{I}}}_{3}+2\mu \bar{\bar{\varepsilon }}$, where λ and μ are the *Lamé coefficients*, ${\bar{\bar{I}}}_{3}$ is the 3×3 unit tensor, and $\bar{\bar{\varepsilon }}\equiv \left(\nabla \vec{u}+\nabla {\vec{u}}^{⊤}\right)/2$ is the *strain tensor*, with the superscript ${}^{⊤}$ standing hereinafter for the transpose. Substituting these into the usual elastodynamic equation, it is written in terms of the displacement vector as

(3) $$\rho \frac{\partial^{2}\vec{u}}{\partial t^{2}}-\nabla \left(\lambda \nabla \cdot \vec{u}\right)-\nabla \cdot \left[\mu \left(\nabla \vec{u}+\nabla {\vec{u}}^{⊤}\right)\right]=\left[\delta \left(z-\frac{h(x,y)}{2}\right)-\delta \left(z+\frac{h(x,y)}{2}\right)\right]\vec{f},$$

where *t* denotes the time variable, ρ is the density, δ is the Dirac delta function, and the two-dimensional component of the forcing term, $\vec{f}$, depends on *x*, *y*, and *t*. In fact, $\vec{f}\left(x,y,t\right)$ will be defined below for the various forcing conditions considered in this paper. Note the appearance of the Dirac delta function, which is due to the fact that, for convenience, the forcing term in (3) is defined as volumetric forcing, while the actual physical forcing will be applied at the lower and upper sides of the plate. The combination δ(z−h(x,y)/2)−δ(z+h(x,y)/2) imposes that forcing is symmetric in *z*.

Equation (3) applies in the spatial domain Ω defined in (1) and must be complemented with the boundary conditions at the boundary of the plate, defined in (2), which impose zero stress, namely

(4) $$\begin{array}{c} \mu \left(\nabla \vec{u}+\nabla {\vec{u}}^{⊤}\right)\cdot \vec{n}+\lambda \left(\nabla \cdot \vec{u}\right)\vec{n}=\vec{0}\;\;\mathrm{at}\;\partial \Omega , \end{array}$$

$\vec{n}$ being the outward unit normal to ∂Ω. In addition, six more scalar boundary conditions at three isolated points of the boundary have to be imposed to eliminate rigid-body motions, but these are not depicted in (4) for the sake of brevity.

As the solution to the problem Equations (3) and (4) is linear on $\vec{f}$, the forcing term appearing in Equation (3), considering a multi-frequency excitation (with *K* different frequencies), produced by *M* forcing devices, can be written as

(5) $$\vec{f}\left(x,y,t\right)=\sum_{k=1}^{K}\sum_{m=1}^{M}{\vec{F}}_{km}\left(x,y\right)\cos\left(\omega_{k}t+\phi_{k}\right),$$

and the dynamics of the resulting displacement vector can be set into the form

(6) $$\vec{u}\left(x,y,z,t\right)=\sum_{k=1}^{K}\sum_{m=1}^{M}{\vec{U}}_{km}\left(x,y,z\right)\cos\left(\omega_{k}t+\phi_{k}\right).$$

Substituting into Equations (3) and (4) expressions Equations (5) and (6), and setting the various coefficients of $\cos(\omega_{k}t+\phi_{k})$ to zero, yields the following (purely spatial) problems (for k=1,…,K and m=1,…,M)

(7) $$\begin{array}{c} \rho \omega_{k}^{2}{\vec{U}}_{km}+\nabla \left(\lambda \nabla \cdot {\vec{U}}_{km}\right)+\nabla \cdot \left[\mu \left(\nabla {\vec{U}}_{km}+\nabla {\vec{U}}_{km}^{⊤}\right)\right]=\\ \;-\left[\delta \left(z-\frac{h(x,y)}{2}\right)-\delta \left(z+\frac{h(x,y)}{2}\right)\right]{\vec{F}}_{km}\left(x,y\right), \end{array}$$

in the spatial domain Equation (1), with the boundary conditions

(8) $$\begin{array}{c} \mu \left(\nabla {\vec{U}}_{km}+\nabla {\vec{U}}_{km}^{⊤}\right)\cdot \vec{n}+\lambda \left(\nabla \cdot {\vec{U}}_{km}\right)\vec{n}=\vec{0}\;\mathrm{at}\;\partial \Omega . \end{array}$$

Now, if forcing terms ${\vec{F}}_{km}$ appearing in Equation (5) were all real, Equations (7) and (8) show that the displacement fields ${\vec{U}}_{km}$ will be also real and each monochromatic component appearing in expansion Equation (6) will represent a standing wave. However, depending on the frequencies $\omega_{k}$ and the phases $\phi_{k}$, the whole spatio-temporal pattern Equation (6) may also be a standing wave or a propagative wave. For instance, by applying Fourier transform to the propagative pulses considered in conventional methods, these can be expanded in the form Equations (5) and (6). In the sequel, all computations will be based on the purely spatial problems Equations (7) and (8), which means that the phases $\phi_{k}$ appearing in Equations (5) and (6) will play no role. This makes a significant difference with conventional methods, in which a good selection of the phases is important to obtain good results. This strategy, based on Equations (7) and (8), will be followed for the sake of simplicity in the presentation, though a more general approach that takes the phases into account could improve the obtained results.

### 2.2. Forcing and Sensing Types

Concerning the *forcing term* ${\vec{F}}_{km}$ appearing in (7), this will be of one of the two types:

- *Point localized* (PL) excitation, in which the force is applied in a small region at a given point of the plate planform, $\left(x_{m},y_{m}\right)$. Recall that because of the factor δ(z−h/2)−δ(z+h/2) in the right hand side of Equation (7), the concentrated force is in fact applied at the upper and lower sides of the plate and is equal at both sides (a symmetric excitation). Thus, the forcing term is given by
  (9) $${\vec{F}}_{km}^{\mathrm{PL}}\left(x,y\right)={\vec{a}}_{km}\delta \left(x-x_{m},y-y_{m}\right),$$
  where the in-plane vector ${\vec{a}}_{km}$ is the applied force and δ is the two dimensional Dirac delta function centered at the point of application of the concentrated force. This excitation mimics non-contact actuators.
- *Rotationally symmetric radial* (RSR) excitation, in which the in-plane imposed force is purely radial and rotationally symmetric in a circle of the plate planform with center at a given point $\left(x_{m},y_{m}\right)$ and radius *r*. Again, because of the factor δ(z−h/2)−δ(z+h/2) in the right hand side of Equation (7), the force is applied at the upper and lower sides of the plate and is equal at both sides. Thus, we have
  (10) $${\vec{F}}_{km}^{\mathrm{RSR}}\left(x,y\right)=a_{km}\vec{g}\left(x-x_{m},y-y_{m},r\right),$$
  where the scalar $a_{km}$ is the overall intensity of the applied force and $\vec{g}\left(x-x_{m},y-y_{m},r\right)$ denotes a forcing term consisting in applying a unit force along the outward normals to the circle of radius *r*, centered at $\left(x_{m},y_{m}\right)$. This mimics piezoelectric actuators.

Similarly, the synthetic data produced, that tries to mimic experimental data, is to be collected at *N* sensors $\left(x_{n},y_{n}\right)$ (with n=1,…,N). We will just retain the in-plane components of the displacement vector, thus, hereinafter, we will consider ${\vec{U}}_{km}$ as two-dimensional in-plane vector. The *N* sensors can be of one of the following two types (which are actually the counterparts of the two types of excitation considered above):

- *Point localized* (PL) sensors, where the (in-plane) displacement vectors at $\left(x_{n},y_{n},\pm h/2\right)$ are measured. Recall that these two vectors are equal because forcing is symmetric in *z* and thus, it suffices measuring one of these vectors. This sensors try to mimic laser Doppler sensors and produces two scalar measurements, namely, the two components of the displacement vector at the sensor.
- *Rotationally symmetric radial* (RSR) sensors in two in-plane circles with centers at $\left(x_{n},y_{n},\pm h/2\right)$ and radius *r*. These sensors are sensitive to the spatial average of the radial (in-plane) displacement vectors along the circles. Again, the measurements at z=±h(x,y)/2 coincide because the three-dimensional displacement vector is symmetric in *z* and, thus, it suffices performing measurements at only one side of the plate. This type of sensors try to mimic the behaviour of piezoelectric sensors of radius *r* centered at $\left(x_{n},y_{n},\pm h/2\right)$ and produce just one scalar measurement each, namely the averaged radial displacement vector.

The election of one or the other forcing and sensing actuators/sensors (be them either PL or RSR) will affect the cost function that will be defined in the following subsection.

According to the description above, the excitation/sensing types are to be combined in Section 3 in three different ways, namely:

(a) PL-forcing and PL-sensing,
(b) RSR-forcing and RSR-sensing,
(c) RSR-forcing and PL-sensing,

considering for each *M* actuator configurations, which will be common to the *K* considered frequencies. For each of these configurations, the synthetic generated measured data will be collected at *N* sensors, being them all of the same type (either PL or RSR). Thus, the available number of different scalar data is K×M×(2N) for PL sensing (where the factor 2 appears because a vector -the surface displacements- measurement is collected at each point) and K×M×N for RSR sensing. However, there are many more unknowns in the inverse problem that are the spatial distributions of ρ, λ, and μ, which take values perturbed from their healthy values just because of the presence of faults in the plate. This involves an *infinite number of scalar unknowns*. Upon discretization via a spatial mesh (with *P* nodes) the number of unknowns is 3P, which is still much larger than the number of available uncorrelated data. This is one of the underlying difficulties of inverse problems. Another, even more relevant, difficulty is that, as anticipated, the (unknown) material properties do not depend continuously on the given data and the inverse problem is generally ill-posed. Obviously, the larger the number of available uncorrelated data, the better for solving the inverse problem. However, we shall roughly maintain the number of forcing/sensing devices and involved frequencies as small as possible, aiming to facilitate future practical implementation of the method.

### 2.3. Cost Function

As anticipated in the introduction, we aim to identify faults in a thin plate by somehow minimizing a cost function that accounts for the difference of measurements in the sensing devices for the damaged plate and the computed counterparts. Specifically, the cost function is defined as

(11) $$H\left(\rho ,\lambda ,\mu \right)=\sum_{k=1}^{K}\sum_{m=1}^{M}\alpha_{km}\int_{\Omega }\left[\delta \left(z-\frac{h}{2}\right)-\delta \left(z+\frac{h}{2}\right)\right]{\vec{H}}_{km}\;dxdydz,$$

where the coefficients $\alpha_{km}>0$ (to be selected later below) measure how every individual contribution of each frequency and each emitting configuration is weighted. The vector functions ${\vec{H}}_{km}$, which depend on the spatial variables and the material parameters, namely

(12) $${\vec{H}}_{km}\left(x,y,z,\rho ,\lambda ,\mu \right),$$

account for the (squared) difference between measurements at the sensing devices and their computed counterparts, and thus depend on the sensing mode. Note the factor δ(z−h/2)−δ(z+h/2) inside the integral in Equation (11) that, as above, comes from the fact that measurements are really performed at z=±h(x,y)/2.

For PL sensing at the points $\left(x_{m1},y_{m1}\right),\ldots ,\left(x_{mN},y_{mN}\right)$, the function ${\vec{H}}_{km}$ is defined as

(13) $${\vec{H}}_{km}^{\mathrm{PL}}=\frac{1}{2}\sum_{n=1}^{N}{\left[{\vec{U}}_{km}^{\mathrm{comput}.}-{\vec{U}}_{km}^{\mathrm{meas}.}\right]}^{2}\delta \left(x-x_{mn},y-y_{mn}\right),$$

where δ is the Dirac delta funcion, ${\vec{U}}^{\mathrm{comput}.}$ is the in-plane component of the computed solution of Equations (7) and (8), and ${\vec{U}}_{km}^{\mathrm{meas}.}$ is its measured counterpart. Note that ${\vec{U}}^{\mathrm{comput}.}$ depends on the spatial variables x,y,z and the parameters ρ,λ,μ and thus ${\vec{H}}_{km}^{\mathrm{PL}}$ exhibits the same dependence, as anticipated in Equation (12). Similarly, for RSR sensing at circles of radius *r* centered at the points $\left(x_{m1},y_{m1}\right),\ldots ,\left(x_{mN},y_{mN}\right)$, we define the function ${\vec{H}}_{km}$ as

(14) $${\vec{H}}_{km}^{\mathrm{RSR}}=\frac{1}{2}\sum_{n=1}^{N}{\langle {\vec{U}}_{km}^{\mathrm{comput}.}-{\vec{U}}_{km}^{\mathrm{meas}.}\rangle }_{x_{mn},y_{mn},r}^{2}\vec{g}\left(x-x_{mn},y-y_{mn},r\right),$$

where ${\vec{U}}^{\mathrm{comput}.}$ and ${\vec{U}}_{km}^{\mathrm{meas}.}$ are as in Equation (13), the rotationally symmetric vector field $\vec{g}$ is as defined after Equation (10), and for a vector field $\vec{U}$, ${\langle \vec{U}\rangle }_{x_{mn},y_{mn},r}$ denotes the average of the in-plane radial component of $\vec{U}$ on the circle of radius *r* centered at the point $\left(x_{mn},y_{mn}\right)$.

Faults in the plate produce changes in the three-dimensional distributions of the density and the Lamé coefficients, which are perturbed as

(15) $$\rho =\rho_{0}-\tilde{\rho },\;\lambda =\lambda_{0}-\tilde{\lambda },\;\mu =\mu_{0}-\tilde{\mu },$$

where the subscript ${}_{0}$ denotes hereinafter the values of the coefficients for the healthy plate and the minus sign comes from the fact that λ, μ, and ρ are usually smaller at the defects than for the healthy plate. If the faults are localized, which is the usual situation, the perturbations are such that

(16) $$∥\tilde{\rho }{∥}_{L_{2}}\ll ∥\rho_{0}{∥}_{L_{2}},\;∥\tilde{\lambda }{∥}_{L_{2}}\ll ∥\lambda_{0}{∥}_{L_{2}},\;∥\tilde{\mu }{∥}_{L_{2}}\ll {∥\mu_{0}∥}_{L_{2}}.$$

where the $L_{2}$-norm of, for example, $\tilde{\rho }$ is defined as

(17) $$∥\tilde{\rho }{∥}_{L_{2}}=\sqrt{\int_{\Omega }{\left|\tilde{\rho }\right|}^{2}\;dxdydz}.$$

It is to be remarked that, although perturbations in the coefficient values need not be small in the defects themselves, conditions Equation (16) still hold because defects are localized. In this case, Equation (16) just implies that the area occupied by the defects must be small compared to the area of the plate, but not that perturbations need to be small. For instance, a circular defect having a diameter of the order of one tenth of either plate sides, has an area that is of the order of one hundredth of that of the plate.

### 2.4. Sensitivities of the Cost Function to Material Properties Perturbations

To proceed further, we consider the sensitivities of the cost function Equation (11) under the perturbations Equation (15) of the material properties, which are defined such that

(18) $$H-H^{0}≃\sum_{m=1}^{M}\sum_{k=1}^{K}\alpha_{km}\int_{\Omega }\left(S_{km}^{\rho }\tilde{\rho }+S_{km}^{\lambda }\tilde{\lambda }+S_{km}^{\mu }\tilde{\mu }\right)\;dxdydz,$$

where $H^{0}$ is the unperturbed value of H, higher order terms (from the quadratic ones) in the small quantities $\tilde{\lambda }$, $\tilde{\mu }$ and $\tilde{\rho }$ have been neglected, and the weights $\alpha_{km}$ coincide with their counterparts in Equation (11).

It turns out that the sensitivities are given by

(19) $$\begin{array}{c} S_{km}^{\rho }=-\omega_{k}^{2}{\vec{U}}_{km}^{\mathrm{dir}.}\cdot {\vec{U}}_{km}^{\mathrm{adj}.},\;S_{km}^{\lambda }=\left(\nabla \cdot {\vec{U}}_{km}^{\mathrm{dir}.}\right)\left(\nabla \cdot {\vec{U}}_{km}^{\mathrm{adj}.}\right), \end{array}$$

(20) $$\begin{array}{c} S_{km}^{\mu }=\frac{1}{2}\left(\nabla {\vec{U}}_{km}^{\mathrm{dir}.}+\nabla {\vec{U}}_{km}^{\mathrm{dir}.⊤}\right):\left(\nabla {\vec{U}}_{km}^{\mathrm{adj}.}+\nabla {\vec{U}}_{km}^{\mathrm{adj}.⊤}\right), \end{array}$$

where: denotes the doubly contracted product of tensors and ${\vec{U}}_{km}^{\mathrm{dir}.}$ and ${\vec{U}}_{km}^{\mathrm{adj}.}$ are the solutions of the *unperturbed direct and adjoint problems*, defined as

$$\begin{array}{c} \rho_{0}\omega_{k}^{2}{\vec{U}}_{km}^{\mathrm{dir}.}+\nabla \left(\lambda_{0}\nabla \cdot {\vec{U}}_{km}^{\mathrm{dir}.}\right)+\nabla \cdot \left[\mu_{0}\left(\nabla {\vec{U}}_{km}^{\mathrm{dir}.}+\nabla {\vec{U}}_{km}^{\mathrm{dir}.⊤}\right)\right]= \end{array}$$

(21) $$\begin{array}{c} \;-\left[\delta \left(z-\frac{h}{2}\right)-\delta \left(z+\frac{h}{2}\right)\right]{\vec{F}}_{km}^{\mathrm{dir}.}\left(x,y\right), \end{array}$$

(22) $$\begin{array}{c} \lambda_{0}\left(\nabla \cdot {\vec{U}}_{km}^{\mathrm{dir}.}\right)\vec{n}+\mu_{0}\left(\nabla {\vec{U}}_{km}^{\mathrm{dir}.}+\nabla {\vec{U}}_{km}^{\mathrm{dir}.⊤}\right)\cdot \vec{n}=\vec{0}\;\mathrm{at}\;\partial \Omega , \end{array}$$

which is precisely the direct problem Equations (7) and (8) for the healthy plate, and

$$\begin{array}{c} \rho_{0}\omega_{k}^{2}{\vec{U}}_{km}^{\mathrm{adj}.}+\nabla \left(\lambda_{0}\nabla \cdot {\vec{U}}_{km}^{\mathrm{adj}.}\right)+\nabla \cdot \left[\mu_{0}\left(\nabla {\vec{U}}_{km}^{\mathrm{adj}.}+\nabla {\vec{U}}_{km}^{\mathrm{adj}.⊤}\right)\right]= \end{array}$$

(23) $$\begin{array}{c} \;-\left[\delta \left(z-\frac{h}{2}\right)-\delta \left(z+\frac{h}{2}\right)\right]{\vec{F}}_{km}^{\mathrm{adj}.}\left(x,y\right), \end{array}$$

(24) $$\begin{array}{c} \lambda_{0}\left(\nabla \cdot {\vec{U}}_{km}^{\mathrm{adj}.}\right)\vec{n}+\mu_{0}\left(\nabla {\vec{U}}_{km}^{\mathrm{adj}.}+\nabla {\vec{U}}_{km}^{\mathrm{adj}.⊤}\right)\cdot \vec{n}=\vec{0}\;\mathrm{at}\;\partial \Omega . \end{array}$$

In the unperturbed direct and adjoint problems, $\lambda_{0}$, $\mu_{0}$, and $\rho_{0}$ are the unperturbed values of the Lamé coefficients and the density, as above, and the forcing terms $F_{km}^{\mathrm{dir}.}$ and $F_{km}^{\mathrm{adj}.}$ depend on the forcing and sensing types, respectively. For the unperturbed direct problem, Equations (21) and (22), ${\vec{F}}_{km}^{\mathrm{dir}.}$ is given by Equations (9) and (10) for PL and RSR forcing, respectively. On the other side, for the unperturbed adjoint problem, Equation (23) and (24), ${\vec{F}}_{km}^{\mathrm{adj}.}$ is given by

(25) $${\vec{F}}_{km}^{\mathrm{adj}.}=\sum_{n=1}^{N}\left[{\vec{U}}_{km}^{\mathrm{dir}.}-{\vec{U}}_{km}^{\mathrm{meas}.}\right]\delta \left(x-x_{mn},y-y_{mn}\right)$$

for PL sensing and

(26) $${\vec{F}}_{km}^{\mathrm{adj}.}=\sum_{n=1}^{N}{\langle {\vec{U}}_{km}^{\mathrm{dir}.}-{\vec{U}}_{km}^{\mathrm{meas}.}\rangle }_{x_{mn},y_{mn},r}\vec{g}\left(x-x_{mn},y-y_{mn},r\right)$$

for RSR sensing. Here, δ, the azimutal average 〈·〉, and the function *g* are as defined after Equations (13) and (14).

Let us now justify Equations (19) and (20) by first perturbing the displacement vector around its value for the healthy plate, as

(27) $${\vec{U}}_{km}={\vec{U}}_{km}^{\mathrm{dir}.}+{\vec{\tilde{U}}}_{km},$$

where ${\vec{U}}_{km}^{\mathrm{dir}.}$ is the solution of the unperturbed direct problem (which corresponds to the healthy plate). Substituting Equations (15) and (27) into Equations (7) and (8) and linearizing yields

$$\begin{array}{c} \omega_{k}^{2}\left(\rho_{0}{\vec{\tilde{U}}}_{km}-\tilde{\rho }{\vec{U}}_{km}^{\mathrm{dir}.}\right)+\nabla \left(\lambda_{0}\nabla \cdot {\vec{\tilde{U}}}_{km}-\tilde{\lambda }\nabla \cdot {\vec{U}}_{km}^{\mathrm{dir}.}\right)+ \end{array}$$

(28) $$\begin{array}{c} \;\nabla \cdot \left[\mu_{0}\left(\nabla {\vec{\tilde{U}}}_{km}+\nabla {\vec{\tilde{U}}}_{km}^{⊤}\right)-\tilde{\mu }\left(\nabla {\vec{U}}_{km}^{\mathrm{dir}.}+\nabla {\vec{U}}_{km}^{\mathrm{dir}.⊤}\right)\right]=0,\\ \left(\lambda_{0}\nabla \cdot {\vec{\tilde{U}}}_{km}-\tilde{\lambda }\nabla \cdot {\vec{U}}_{km}^{\mathrm{dir}.}\right)\vec{n}+\mu_{0}\left(\nabla {\vec{\tilde{U}}}_{km}+\nabla {\vec{\tilde{U}}}_{km}^{⊤}\right)\cdot \vec{n}- \end{array}$$

(29) $$\begin{array}{c} \;\tilde{\mu }\left(\nabla {\vec{U}}_{km}^{\mathrm{dir}.}+\nabla {\vec{U}}_{km}^{\mathrm{dir}.⊤}\right)\cdot \vec{n}=\vec{0}\;\mathrm{at}\;\partial \Omega . \end{array}$$

Similarly, substituting Equations (15) and (27) into Equation (11), invoking Equations (13) and (14), and linearizing lead to

(30) $$H-H_{0}≃\sum_{k=1}^{K}\sum_{m=1}^{M}\alpha_{km}\int_{\Omega }\left[\delta \left(z-\frac{h}{2}\right)-\delta \left(z+\frac{h}{2}\right)\right]{\vec{\tilde{H}}}_{km}\;dxdydz,$$

where the vector function ${\vec{\tilde{H}}}_{km}$ is given by

(31) $${\vec{\tilde{H}}}_{km}^{\mathrm{PL}}=\sum_{n=1}^{N}{\vec{\tilde{U}}}_{km}\cdot \left[{\vec{U}}_{km}^{d}-{\vec{U}}_{km}^{\mathrm{meas}.}\right]\delta \left(x-x_{mn},y-y_{mn}\right),$$

and

(32) $${\vec{\tilde{H}}}_{km}^{\mathrm{RSR}}=\sum_{n=1}^{N}{\vec{\tilde{U}}}_{km}\cdot {\langle {\vec{U}}_{km}^{\mathrm{dir}.}-{\vec{U}}_{km}^{\mathrm{meas}.}\rangle }_{x_{mn},y_{mn},r}\vec{g}\left(x-x_{mn},y-y_{mn},r\right),$$

for PL and RSR sensing, respectively. Thus, invoking Equations (25) and (26), yields, for both PL and RSR sensing,

(33) $${\vec{\tilde{H}}}_{km}={\vec{F}}_{km}^{\mathrm{adj}.}\cdot {\vec{\tilde{U}}}_{km},$$

where ${\vec{F}}_{km}^{\mathrm{adj}.}$ is the forcing term in the adjoint Equation (23).

To proceed, we multiply (with the usual Euclidean inner product) Equation (28) by the solution of the adjoint problem Equations (23) and (24), subtract to this equation the product of Equation (23) by ${\vec{\tilde{U}}}_{km}$, integrate in the domain occupied by the plate, integrate by parts, and invoke the boundary conditions (24) and (29). After some algebra, we obtain

(34) $$\begin{array}{c} \int_{\Omega }\left[-\omega_{k}^{2}\;\tilde{\rho }\;{\vec{U}}_{km}^{\mathrm{dir}.}\cdot {\vec{U}}_{km}^{\mathrm{adj}.}+\tilde{\lambda }\left(\nabla \cdot {\vec{U}}_{km}^{\mathrm{dir}.})(\nabla \cdot {\vec{U}}_{km}^{\mathrm{adj}.}\right)\right]\;dxdydz+\\ \;\frac{1}{2}\int_{\Omega }\tilde{\mu }\left(\nabla {\vec{U}}_{km}^{\mathrm{dir}.}+\nabla {\vec{U}}_{km}^{\mathrm{dir}.⊤}\right):\left(\nabla {\vec{U}}_{km}^{\mathrm{adj}.}+\nabla {\vec{U}}_{km}^{{\mathrm{adj}.}_{⊤}}\right)\;dxdydz=\\ \;\int_{\Omega }\left[\delta \left(z-\frac{h}{2}\right)-\delta \left(z+\frac{h}{2}\right)\right]{\vec{F}}_{km}^{\mathrm{adj}.}{\vec{\tilde{U}}}_{km}\;dxdydz. \end{array}$$

This equation, in conjunction with Equations (18), (30), and (33), readily leads to the expressions Equations (19) and (20), thus completing the derivation of these equations.

### 2.5. Topological Derivative and Its Numerical Computation

Once we have computed the (three-dimensional) sensitivities of the cost function, defects are expected at those points where the sensitivities peak. However, we have three sensitivities, which could peak at different points, and it is needed to combine the sensitivities. Here, we combine them as in Reference [38]

(35) $$T_{km}=\rho S_{km}^{\rho }+\frac{1}{\mu (1+\nu )}\left[\frac{\nu -1-2\nu^{2}}{2(1-\nu^{2})}S_{km}^{\lambda }+S_{km}^{\mu }\right],$$

where ν=λ/[2(λ+μ)] is the Poisson ratio, and consider the overall sensitivity

(36) $$T=\sum_{k=1}^{K}\sum_{m=1}^{M}\alpha_{km}T_{km}.$$

The weights $\alpha_{km}$ are the counterparts of those appearing in Equation (11). Equations (35) and (36) correspond to the so called *topological derivative*, which can also be defined at each point of Ω as follows [26,27,28]. We consider a small spherical fault, with center at (x,y) and radius *r*. The topological derivative can be defined as the limit

(37) $$T\left(x,y,z\right)=\lim_{r\to 0}\frac{H^{r}-H^{0}}{4\pi r^{3}/3},$$

where $H^{r}$ and $H^{0}$ are the values of the cost function Equation (11) for the damaged and healthy plates, respectively, and $4\pi r^{3}/3$ is the volume of the spherical fault. Thus, the topological derivative at (x,y,z) somehow measures the sensitivity of the objective function to an infinitesimal defect located at a given point. Although we shall use Equation (35) to estimate faults along the paper, it is to be noted that the sensitivities could be combined in other ways. However, this extension is well beyond the scope of this paper.

Finally, we must define the weights appearing in Equations (11) and (36). Following some ideas found in Reference [33], which gave very good results for simply analyzing the two-dimensional wave equation and also in simpler SHM problems [30], we select

(38) $$\alpha_{km}=\frac{1}{\left|\min_{\left(x,y,z\right)\in \Omega_{I}}T_{km}\left(x,y,z\right)\right|},$$

where the *interrogation window* $\Omega_{I}$ is a strict subset of the plate, defined as (cf Equation (1))

(39) $$\Omega_{I}:\left(x,y\right)\in \Gamma_{I},\;-\frac{h(x,y)}{2}\leq z\leq \frac{h(x,y)}{2}$$

with $\Gamma_{I}\subset \Gamma$. In fact, $\Omega_{I}$ is the subset of the plate where the topological derivative will be calculated (the interrogation window). It must exclude a vicinity of the actuators and sensors as the topological derivative would take very large (positive or negative) values near these points. This is because both the solutions to the unperturbed direct and adjoint problems are very steep near the actuators and sensors, respectively, and the topological derivative includes terms that are proportional to the product of the gradients of the unperturbed direct and adjoint problems yielding larges values of the topological derivative as we approach to either actuators or sensors. The large values of the topological derivative near these points could give negative peaks that would be spurious in the present context and will be avoided.

Now, we invoke either Equations (18)–(20) or (37) and note that the actual faults are expected such that the objective function decreases. Thus, the location of the faults is labeled by negative peaks of the topological derivative.

Summarizing the above, the topological derivative is computed in the interrogation window Equation (39) using Equations (35), (36), and (38), where the sensitivities are given by Equations (19) and (20). The latter equations involve the solutions of the unperturbed direct and adjoint problems, Equations (21) and (22) and (23)–(24), respectively. Both problems are numerically solved in this paper using the ANSYS Mechanical FEM solver [63] with a second degree polynomial as shape function (the element SOLID186). Note that the material properties in both the unperturbed direct and adjoint problems are just those for the healthy plate. If experimental data were available, these would be used in the forcing terms of the adjoint problems Equations Equations (23) and (24) and the computation of the topological derivative would be completed. However, in our case, we will also compute synthetic data. This will be done solving the full (perturbed) direct problem Equations (7) and (8), where the material properties are the actual ones, accounting for the presence of faults. This latter problem will be solved using the same ANSYS FEM solver mentioned above, but with a different mesh to avoid inverse crimes (see below). Details on the meshes used to solved these problems will be given in the next section.

## 3. Results

As an application example of the method, we consider an aluminium plate, with constant density ρ=2700 Kg/m${}^{3}$ and Lamé coefficients λ=50.35 GPa and μ=25.94 GPa (Young modulus E=69 GPa and Poisson ratio ν=0.33). We use the Cartesian coordinate system anticipated in Section 2.1.

**Figure 1:**
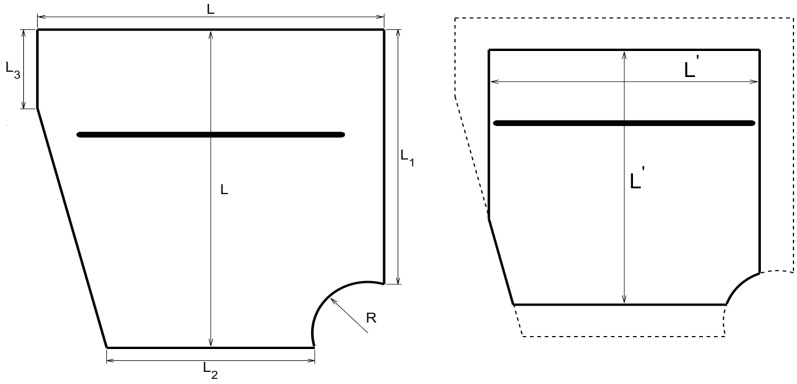
(**Left**): sketch of the complex planform of the plate. (**Right**): planform of the associated interrogation window (solid line) included in the plate (dashed line).

Two types of healthy plates will be dealt with:

- Rectangular plates with variable thickness, which have been considered in connection with the simulation of guided waves in Reference [64], where comparison with experimental results has also been made. In the present paper, the planform Γ of the plates with variable thickness will always be a 50×50 cm${}^{2}$ square, while the planform of the interrogation window $\Gamma_{I}$ will be a 40×40 cm${}^{2}$ centered square. Concerning the thickness, this will vary from 1 mm to 2.5 mm in various ways, from the emitting/sensing side of the plate to the opposite side. Namely, thin/thick/thin, thick/thin/thick, thin/thick, and thick/thin. In all cases, the thickness variation will be fairly sharp.
- Plates with a more complex planform, which will be as sketched in Figure 1-left, where L=50 cm, $L_{1}=40$ cm, $L_{2}=30$ cm, $L_{3}=12.7$ cm, and R=0.8 cm; the interrogation window will be as sketched in Figure 1-right, where $L^{\prime }=40$ cm. Concerning the thickness of this plate, this is h=1 mm. Moreover, in order to increase the complexity of the configuration of the healthy plate, an elongated, centered through slit will be added parallel to the plate side with length *L*, at a distance 16.5 cm to this side of the plate. The slit length and width will be 38.8 cm and 7.88 cm, respectively.

Concerning the emitting/sensing configurations, as anticipated in Section 2.2, these will be of one of the following types, namely: PL emitting/PL sensing, RSR emitting/RSR sensing, and RSR emitting/PL sensing. To obtain comparable results for the various considered cases, the actuators and sensors will always be located at 1.5 cm distance from a straight boundary of the plate (the same part of the boundary for both emitting and sensing devices). This small 1.5 cm distance has been selected on purpose to be very small compared to the plate size and would lead to strong difficulties if other standard SHM methods were used. No particular care will be taken in choosing the location of the actuators and sensors along the line parallel to the involved side of the plate to emphasize robustness of the method. RSR actuators and sensors will always be circular, with a r=5 mm radius (similar to the radius of typical piezo-electric devices).

The defects will always be placed inside the interrogation window $\Omega_{I}$ and will be circular through-holes, with a small size, namely 2.5 mm in radius. For the sake of clarity, the topological derivative will be plotted in the mid plane of the interrogation window, $\Gamma_{I}$. In fact, since it is the (negative) peaks of the topological derivative that matters to localize defects, the topological derivative will be always rescaled as follows. The topological derivative outside $\Gamma_{I}$ will be set to zero and the positive values of the topological derivative inside $\Gamma_{I}$ will also be replaced by zero. The negative values of the topological derivative inside $\Gamma_{I}$ will be rescaled with the maximum of its absolute value inside $\Gamma_{I}$. Following this procedure, all topological derivatives are rescaled in the range between 0 and −1.

The amount of available data can be increased by either increasing the number of sensors, *N*, the number of actuators/sensors configurations, *M* or the number of involved frequencies, *K*. However, increasing *N* and/or *M* requires increasing the number of installed devices. Instead, increasing *K*, which only affects the complexity of the emitted signal, according to (5) is much simpler. Thus, some emphasis will be put below in decreasing *N* and *M* (at the expense of increasing *K*). Based on our previous experience [30], the number of emitting/sensing devices will always be both limited, namely equal to four for PL receivers and eight for RSR receivers. Although the number of devices could be increased in our method with no difficulties, this will not be done for the sake of emphasize robustness of the method. It also has to be considered that, placing actuators/sensors that are somewhat close to each other (which makes the available data less uncorrelated) and close to the boundary of the plate eases compact setups of the experimental device and implementation.

**Figure 2:**
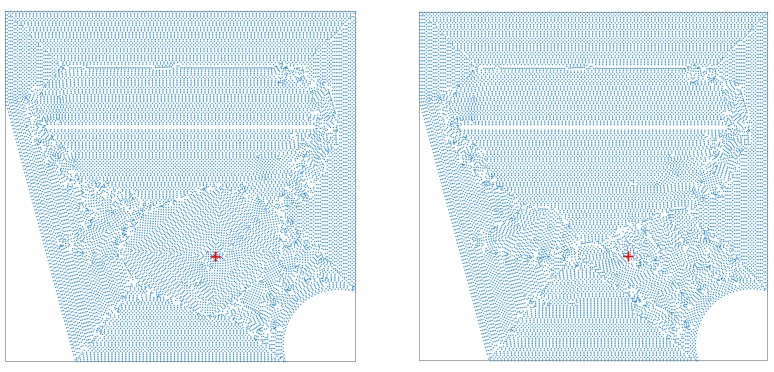
Meshes used to compute the experimental (synthetic) data (**left**) and the solution of the direct and adjoint problems (**right**), The whole amount of nodes (which would lead to too dense plots) is not shown, but only one out each of 4 nodes are shown. The defect location is indicated with a red + symbol in both meshes.

As anticipated in Section 2.5, the application of the method developed in this paper requires solving various elastic problems. Namely, the full problem (7)–(8) (with the actual distributions of ρ, λ, and μ, taking the faults into account) to obtain synthetic (simulated experimental) data, the unperturbed direct problem and the adjoint problem, Equations (21)–(24), respectively, with the material properties equal to their values for the healthy plate. Recall that the adjoint problem forcing term depends on the simulated experimental data. The typical size of the individual elements of the computational mesh along the plate planform will be ∼2.5 mm and just one element throughout thickness. This will give a number of degrees of freedom ∼0.85 Million (corresponding to 0.283 Million nodes). The meshes used to generate the synthetic data and to solve the unperturbed direct and adjoint problems for the sample considered in Figure 1 are given in Figure 2.

It is ease to distinguish that the two meshes are fairly different among each other, both qualitatively and quantitatively. The main qualitative difference between them is that nodes concentrate near the defect in the left plot (because the defect is taken into account when computing synthetic data), but not in the right plot, which corresponds to computations of the unperturbed direct problem and the adjoint problem in the healthy plate. Using different meshes avoids the so called (in the context of inverse problems) *inverse crime*, which occurs when the same numerical ingredients are used to both synthesizing data and solving the inverse problem [40,65,66]). The forcing frequencies will be chosen as equispaced in a range that will be the same for all considered cases, namely

(40) 20kHz≤ω≤40kHz,

to further emphasize the robustness of the method. Note that some of these frequencies can be close to natural frequencies of the plate, which will give disparate amplitudes of the generated waves. However, because of the scaling Equation (38), the effect of these high amplitude waves will not mask the contribution of the remaining waves.

In the remaining of this section, we consider the two anticipated cases, namely a rectangular plate with variable thickness, in Section 3.1, and a plate with complex planform, in Section 3.2.

### 3.1. Rectangular Plate with a Variable Thickness

As anticipated, forcing and sensing devices will be fairly close to the plate boundary, namely 1.5 cm apart in all cases considered below. Also, these devices will be equispaced along the line parallel to the boundary where they are located. When considering RSR devices, their radius will be 5 mm. When performing PL emission, the excitation will be perpendicular to the boundary of the plate that is closest to the emitters. Also, in all cases, the considered fault will be a through-hole circular defect of radius 2.5 mm, centered at the point whose coordinates are (x,y)=(0.05m,−0.1m) in a Cartesian coordinate system with its origin at the center of the mid-section of the plate. The defect will be indicated with a white cross.

In the remaining of this subsection, we consider several distributions of the thickness of the plate.

#### 3.1.1. Thin-Thick-Thin Thickness Distribution

**Figure 3:**
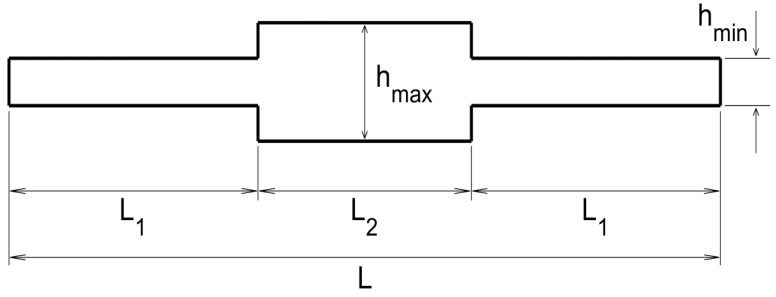
Sketch of the thin-thick-thin thickness distribution along the lateral section of the plate.

The sketch of the transversal section of the plate, from the emitting/sensing side to the opposite side, is as sketched in Figure 3.

The various lengths appearing in this sketch are L=50 cm, $L_{1}=17.5$ cm, $L_{2}=15$ cm, $h_{\min}=1$ mm, and $h_{\max}=2.5$ mm. As can be seen in this figure, the thickness variation is very sharp, which represents a difficulty for standard methods due to reflection/refraction at the sharp edges. An additional difficulty is the different wave propagation velocity at those parts of the plate exhibiting different thickness.

**Figure 4:**
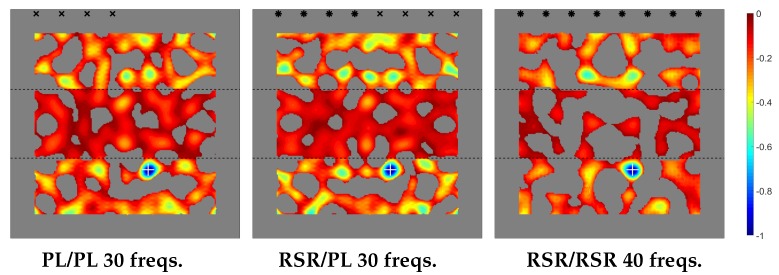
Thin-thick-thin thickness distribution. Topological derivatives with different types of emission/sensing devices.

The plot of the topological derivative for the various emitting/receiving configurations is given in Figure 4, where the sharp thickness increase/decrease is indicated with horizontal lines. Also, the location of the emitting/receiving devices is indicated as follows.

Dual PL/PL emitting/sensing devices (in the left plot) are marked with black ×-symbols and dual RSR-RSR devices (in the right plot), with black asterisks. Consistently, in the middle plot, RSR emitters are labeled with black asterisks and PL receivers, with black ×-symbols. This convention will be followed in all plots of the topological derivative along the remaining of the paper.

Our prediction of the position of the defects is where negative peaks of the topological derivative are located. This corresponds to the small blue region in Figure 4, where the actual position of the defect is also indicated with a white cross. As can be seen, the topological derivative locates the defect quite well. In fact, the negative peak of the topological derivative is extremely close to the position of the defect, which cannot be seen in the figure because of the plot is not precise enough. This will occur with the remaining cases considered in this paper.

The results in Figure 4 are consistent with our comments above about the efficiency of the method, which provides better results in the PL/PL emitting/receiving configuration, since only four devices and 30 frequencies are needed. The method works equally well for the RSR/PL configuration, since four emitters and four receivers are again needed, and 30 frequencies are involved. The only difference is that emitters and receivers are of different type and thus located at different positions. Instead, the performance of the method worsens in the RSR/RSR emitting/receiving configuration, since, to obtain results that are comparable to their counterparts in the former two cases, (a) the number of emitters and receivers must be doubled, and (b) the number of frequencies increased. This is because RSR sensing essentially halves the number of receiving data compared to PL sensing, namely one scalar instead of a vector at each sensor for each frequency.

Finally, Figure 4 also shows that the absolute value of the topological derivative is smaller in the thick part of the plate (namely, in that region in between of the two parallel lines) than in the thin part of the plate. The reason is that the solutions of the adjoint and unperturbed direct problems are smaller in the thick part of the plate, where the energy of the waves per unit area of the plate planform is also smaller.

#### 3.1.2. Thick-Thin-Thick Thickness Distribution

**Figure 5:**
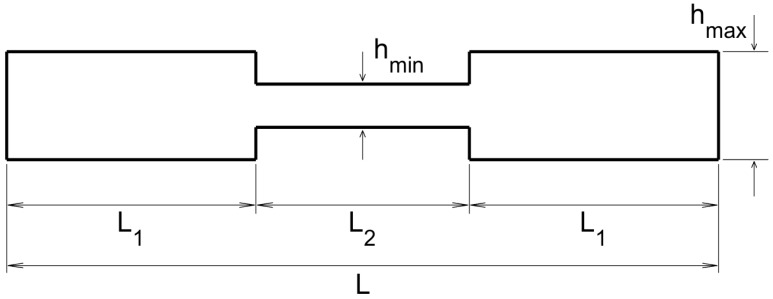
Counterpart of Figure 3 for the thick-thin-thick thickness distribution.

This is the dual case of that considered in the last sub-subsection. Now, the transversal section of the plate, from the emitting/sensing side to the opposite side, is as sketched in Figure 5, where the various indicated lengths are L=50 cm, $L_{1}=17.5$ cm, $L_{2}=15$ cm, $h_{min}=1$ mm, and $h_{max}=2.5$ mm. Note that, as in Figure 3, the plate exhibits sharp thickness variations, which again will make very difficult to use standard methods.

**Figure 6:**
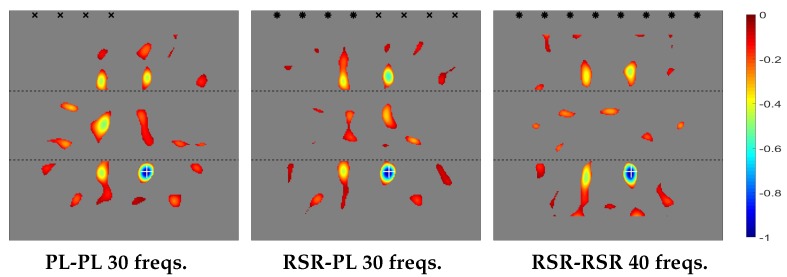
Counterpart of Figure 4 for the thick-thin-thick thickness distribution.

The topological derivative for the present case (considering the same emitters/sensors configurations as in the fomer case) is given in Figure 6. As can be seen, the results are fairly good and the performance of the method follows a similar trend as in the previous case. Namely, the application of the method requires the same number of devices and the same number of involved frequencies as in the previous case. And, again, using PL/PL and RSR/PL emitters/receivers distributions, the number of emitting/receiving devices and the number of required frequencies are both smaller than their counterparts using the RSR/RSR configuration.

#### 3.1.3. Thin-Thick Thickness Distribution

**Figure 7:**
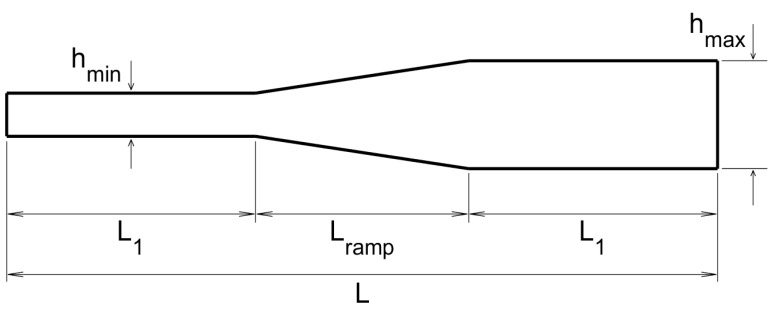
Counterpart of Figure 3 for the thin-thick thickness distribution.

The transversal section of the plate in this case, from the emitting/receiving side to the opposite side, is as sketched in Figure 7, where the various indicated lengths are L=50 cm, $L_{1}=22.5$ cm, $L_{\mathrm{ramp}}=5$ cm, $h_{\min}=1$ mm, and $h_{\max}=2.5$ mm. Note that the thickness variation is not sharp in the present case, but is imposed along a short ramp of length $L_{\mathrm{ramp}}$. Also, the emitters and the receivers are located in the thin side of the plate.

**Figure 8:**
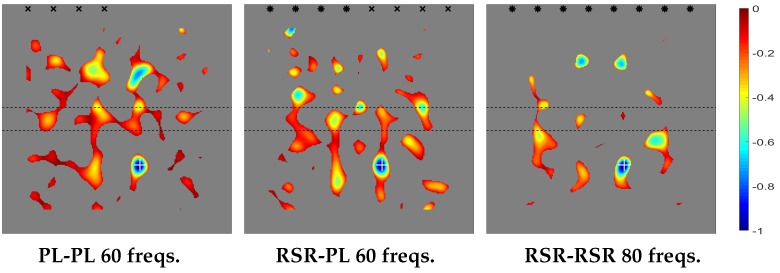
Counterpart of Figure 4 for the thin-thick thickness distribution.

The topological derivative for the present case is given in Figure 8, where it can be seen that the method performs well and shows similar trends as in the previous cases in connection with the emitters/receivers configurations. However, the number of required frequencies is now larger. This is because the defect is located in the thick part of the plate, where according to our previous comment on Figure 4, the contrast in the topological derivative is smaller.

#### 3.1.4. Thick-Thin Thickness Distribution

**Figure 9:**
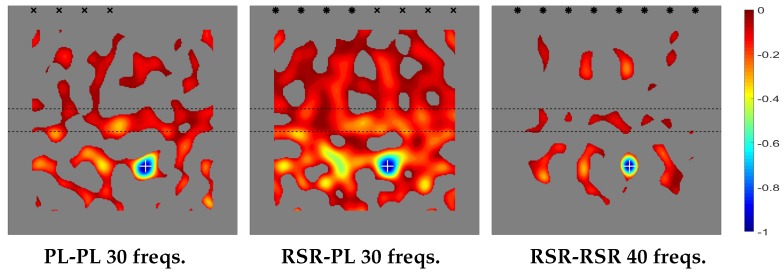
Counterpart of Figure 4 for the thick-thin thickness distribution.

The lateral section of the plate in this case is the same as in the latest case, sketched in Figure 7, except that now the emitters and receivers are located in the thick side of the plate. The topological derivative is given in Figure 9. Comparison with Figure 8 shows that the method gives comparable results as in the former case using a smaller number of frequencies. This is due to the fact that now the defect is located in the thin part of the plate, where the contrast in the topological derivative is larger.

### 3.2. Complex Planform Plate

Let us now consider the plate with a more complex planform sketched in Figure 1, which exhibits a through slit sketched in that figure, and considering the interrogation window also sketched in that figure. The emitting/sensing device configurations are as in the last section and located 1.5 cm apart from that part of the boundary with length *L*. As in the previous cases, the defect to be detected is a small circular through hole of radius 2.5 mm. Its center is located at 35 cm and 20 cm from the upper and right sides of the plate, respectively. Note that the present complex geometry case is very demanding and hardly accessible to standard SHM methods.

**Figure 10:**
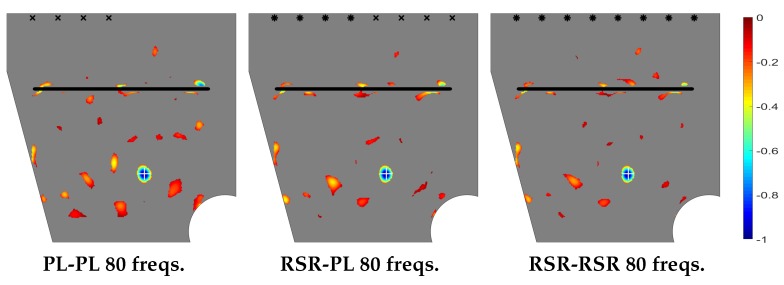
Counterpart of Figure 4 for the plate with complex planform.

The topological derivative for the present case is given in Figure 10, where it can be seen that the method performs quite well in localizing the defect. However, the required frequencies number is larger than in the cases considered in the last section. This is due to the presence of the through slit, which partially hides the defect.

## 4. Conclusions

A method for solving inverse problems based on the multi-frequency topological derivative has been developed for the phenomenon of permanent multifrequency guided waves. The method has been applied to various very demanding problems involving SHM in plates with both:

- Variable thickness in rectangular plates.
- Complex planform that included both a complex shape of the boundary of the plate and a through slit.

In all cases, the method has proven to give fairly good results in localizing the very small faults using a limited number of emitting and sensing devices that, moreover, were very close to the boundary. The various advantages of the method proposed in this paper over conventional methods are due to various reasons:

- Instead of using a single frequency pulse, our method uses a permanent multifrequency excitation, which is easier to produce experimentally.
- By using the non-simplified elasto-dynamic equations, instead of the simplified model of Lamb wave propagation, the mode conversion associated to variable thickness and complex planform is naturally taken into account, without further ad hoc ingredients.
- From the signals received by a discrete number of sensors, the propagating disturbances caused by the existing defects can be identified in the method presented in the paper. This is done by the topological derivative algorithm, which accurately identifies the defects, as it has been demonstrated in the various considered applications.

As a proof of the concept, we have used synthetic data instead of plain experimental data, whose generation involves additional difficulties that are beyond the scope of this paper and are left as the object of future research. In any event, we hope that the results presented in the paper are convincing enough as to conclude that the method is an advantageous alternative to standard SHM methods based on the time of flight for propagative guided waves pulses.
